# Supplementary material for: Synthetic microparticles conjugated with VEGF165 improve the survival of endothelial progenitor cells via microRNA-17 inhibition
Source: Nat Commun. 2017 Sep 29;8:747. doi: 10.1038/s41467-017-00746-7 (PMC5622042; doi:10.1038/s41467-017-00746-7)
Supplement: Supplementary file 1 — Supplementary Information [file 41467_2017_746_MOESM1_ESM.pdf]

SI GUIDE

File Name: Supplementary Information
Description: Supplementary Figures, Supplementary Tables and Supplementary References.

File Name: Supplementary Data 1
Description: Downregulated miRNAs in the microarray

File Name: Supplementary Data 2
Description: Upregulated miRNAs in the microarray

File Name: Peer Review File
Description:

Supplementary Table 1. Antibodies used for experiments

Antibody	Dilution	Reference	Supplier	Fixation
Mouse anti-his tag (biotinylated)	See Text°	BAM050	R&D Systems	N/A
Mouse anti-VEGF	10µl/test°□	IC2931P	R&D Systems	N/A
Mouse anti-VEGF Receptor 2	1/50◆	ab9530	Abcam	4% PFA
Rabbit anti-human p-Flk-1 (Tyr 1175)	1/1000⊙	sc-101819	Santa Cruz	N/A
Rabbit anti-human p-Flk-1 (Tyr 1214)	1/1000⊙	sc-101820	Santa Cruz	N/A
Rabbit anti-CDKN1A	1/50◆□	sc-397	Santa Cruz	4% PFA
Rabbit anti-ZO1	1/200◆	61-7300	Invitrogen	4% PFA
Mouse anti-VE-cadherin	1/50◆	sc-9989	Santa Cruz	4% PFA
Alexa Fluor 555 anti mouse	1/200◆	A21422	Molecular Probes	4% PFA
Alexa Fluor 488 anti rabbit	1/200◆	A11034	Molecular Probes	4% PFA
Cy3 anti rabbit	1/100◆	111-165-144	Jackson Immunoresearch	4% PFA
DAPI	2 µg/ml◆	D9542	Sigma	4% PFA

°Microparticle preparation/characterization (for the amount of antibodies, please, see the text), ◆immunofluorescence, ⊙WB, and □flow cytometry.

Supplementary Table 2. Primers used for quantitative RT-PCR

Gene/RNA	Forward sequence	Reverse sequence
SNORD48	ATGATGACCCCAGGTAACTCT	GCTGCGGTGATGGCAT
5S rRNA	GATCTCGTCTGATCTCGGAAG	GGTATTCCCAGGCGGTCT
hsa-miR-17	CAAAGTGCTTACAGTGCAGGTAG	Universal primer
hsa-miR-217	TACTGCATCAGGAACTGATTGGA	Universal primer
hsa-miR-222	AGCTACATCTGGCTACTGGGT	Universal primer
U6	TCGGCAGCACATATACTAA	GAATTTGCGTGTCATCCT
EIF4G2	GAGCCATTGCTAAGATCAAG	CTAATCTAGGTCCCACTGTC
GRM7	TGAACTCAATGTCCAGAAAC	CTGTTTGGGTCTACGTTTTC
SATL1	GATAGCCATCACAACTCAATG	CTGTTAAACCTGAAGAGATGC
ZNF652	GTTTCAGTACAAGTACCAGC	AGATAAAGGGTTTCTCTCCAG
CCND1	GCCTCTAAGATGAAGGAGAC	CCATTTGCAGCAGCTC
CDKN1A	CAGCATGACAGATTTCTACC	CAGGGTATGTACATGAGGAG
E2F1	CTGATGAATATCTGTACTACGC	CTTTGATCACCATAACCATCTG
S1PR1	CAGACAAGCAAAACAAAGTG	CATCAACAAAAGTGCCAAAG
HSPB1	CTTCACGCGGAAATACAC	ATGGTGATCTCGTTGGAC
JAK1	GAAAAACAAGATCCGGGAAG	TCCATTTTCTTGTTGTCCTG
MAPK9	CCTGAAGATCCTTGACTTTG	ATCAACGTTCTCTTTGTAGC
CASP3	AGCGAATCAATGGACTCT	TTTCTGAATGTTTCCCTGAG
CASP9	TTGTTCATCTCCTGCTTAGA	TCTGGTTTGCGAATCTCT

Supplementary Table 3. The individual results for the top 20 most differentially expressed known mRNA genes detected by mRNA sequencing

Gene ID	Gene	Locus	Ctrl FPKM	Transf FPKM	Log2_FC	q Value
XLOC_046949	FAM65B	6:24797548-25057301	8.542	0.008	-10.0525	0.000675141
XLOC_004640	CD84	1:160506862-160549306	0.038	6.274	7.35534	0.000675141
XLOC_037760	C3orf65	3:185360843-185542844	1.108	0.011	-6.60622	0.0232187
XLOC_038309	CELSR3	3:48673901-48700348	20.027	0.220	-6.50982	0.000675141
XLOC_050299	-	7:64435760-64435947	2.697	134.302	5.63773	0.0165759
XLOC_061341	CDKL5	X:18443702-18690229	1.548	0.037	-5.38025	0.000675141
XLOC_054281	MTND4P15	9:94793357-94877672	0.055	2.100	5.26693	0.0405763
XLOC_031778	-	2:96497268-96500294	0.899	0.025	-5.15123	0.0208843
XLOC_047912	SAMD3	6:130465459-130686570	0.034	1.084	5.00886	0.000675141
XLOC_012270	-	12:105877461-105881171	2.088	0.075	-4.80211	0.000675141
XLOC_053059	-	8:81397172-81397365	2.305	63.537	4.78454	0.0381035
XLOC_036358	GRM7	3:6532165-7783215	0.125	3.287	4.7208	0.000675141
XLOC_012269	-	12:105875536-105877390	1.788	0.070	-4.68055	0.00125545
XLOC_018141	FAM81A	15:59427112-59815748	0.233	0.009	-4.65827	0.000675141
XLOC_018244	GLCE	15:69452922-69564549	903.080	36.124	-4.64382	0.0133201
XLOC_019972	TMC5	16:19421817-19510435	0.992	0.046	-4.43902	0.000675141
XLOC_015426	TMEM253	14:21484921-21572881	0.864	0.040	-4.42957	0.0057667
XLOC_016127	-	14:86101873-86102316	0.182	3.837	4.39655	0.0497288
XLOC_012926	IFLTD1	12:25562226-25801513	1.360	0.065	-4.39061	0.000675141
XLOC_008587	-	11:58345977-58346438	4.933	0.237	-4.38203	0.0278032

Table of the 20 most differentially expressed mRNAs, with log fold change (Log2_FC) between groups with Benjamini-Hochberg FDR corrected q-values. The list is sorted on Log2_FC.

Supplementary Table 4. The individual results for the top 20 most differentially expressed isoforms detected by mRNA sequencing

Isoform ID	Gene	Locus	Ctrl FPKM	Transf FPKM	Log2_FC	q Value
XLOC_019533	ANPEP	15:90328119-90358633	1.34842	1.02E-155	-515.306	0.0399145
XLOC_015291	COL4A1	13:110801317-111165380	106.989	4.68E-137	-459.62	0.0385023
XLOC_031368	EFEMP1	2:56093101-56151274	13.6848	3.17E-130	-433.962	0.0280797
XLOC_009759	EIF4G2	11:10804859-10830657	6.11E-97	88.4983	326.083	0.0494118
XLOC_012686	ANO2	12:5641034-6233936	1.98074	1.03E-82	-273.339	0.0328669
XLOC_037744	PSMD2	3:183852825-184402546	1.80386	4.44E-65	-214.627	0.0344827
XLOC_030854	SEPT2	2:242166678-242293442	1.05888	6.10E-65	-213.4	0.0344827
XLOC_017896	THBS1	15:39873279-39891667	18.9287	1.72E-58	-196.13	0.0265174
XLOC_036170	RPL3	22:39708886-39716394	4.37E-33	1.20503	107.765	0.0216794
XLOC_047558	EEF1A1	6:74225472-74280319	0.919699	3.48E-33	-107.705	0.0344827
XLOC_013317	SMARCC2	12:56544579-56584068	3.13E-25	0.740708	80.9687	0.0494118
XLOC_047558	EEF1A1	6:74225472-74280319	1629.19	1.69E-19	-73.0315	0.0313791
XLOC_017114	LTBP2	14:74964872-75079306	20.9293	1.56E-18	-63.5379	0.0328669
XLOC_009915	CD59	11:33719806-33757991	1.06E-17	66.6759	62.4475	0.0297186
XLOC_034040	TGM2	20:36756858-36794980	1.19E-13	37.1242	48.1503	0.023296
XLOC_017896	THBS1	15:39873279-39891667	441.431	1.46E-09	-38.1407	0.0328669
XLOC_017896	THBS1	15:39873279-39891667	824.637	3.68123	-7.80743	0.0265174
XLOC_033810	RRBP1	20:17594321-17662940	0.220206	47.8513	7.76356	0.0425472
XLOC_003088	UBR4	1:19400999-19536770	11.2597	0.335811	-5.06737	0.00335663
XLOC_012270	-	12:105877461-105881171	2.08817	0.0748493	-4.80211	0.00335663

Table of the 20 most significantly differentially expressed isoforms, with log fold change (Log2_FC) between groups with Benjamini-Hochberg FDR corrected q-values. The list is sorted on Log2_FC.

Supplementary Table 5. The top 20 differentially expressed novel transcripts identified in mRNA sequencing

Novel Transcript ID	Closest Related Known Gene	Locus	Ctrl	Transf FPKM	Log2_FC	q Value
TCONS_00085720	COL4A1	13:110801317-111165380	106.989	4.6E-137	-459.62	0.0385023
TCONS_00052415	EIF4G2	11:10804859-10830657	6.11E-97	88.4983	326.083	0.0494118
TCONS_00075943	SMARCC2	12:56544579-56584068	3.13E-25	0.740708	80.9687	0.0494118
TCONS_00205680	TGM2	20:36756858-36794980	1.19E-13	37.1242	48.1503	0.023296
TCONS_00099624	THBS1	15:39873279-39891667	824.637	3.68123	-7.80743	0.0265174
TCONS_00204501	RRBP1	20:17594321-17662940	0.220206	47.8513	7.76356	0.0425472
TCONS_00085429	ABCC4	13:95672082-95983687	3.14261	0.159508	-4.30026	0.00335663
TCONS_00124039	TBC1D10B	16:30368421-30381585	1.43246	26.6936	4.21992	0.00335663
TCONS_00206441	ATP9A	20:50213052-50385173	10.181	0.595275	-4.09619	0.0164573
TCONS_00032986	SCD	10:102106876-102124591	66.1694	4.37847	-3.91766	0.0164573
TCONS_00128759	XAF1	17:6658765-6678989	1.87214	24.5972	3.71573	0.0164573
TCONS_00122809	SMG1	16:18814404-18937776	5.37056	0.411391	-3.70649	0.00335663
TCONS_00231057	TMEM115	3:50384760-50397041	2.25671	27.4838	3.60629	0.0344827
TCONS_00228488	RFTN1	3:16306699-16555533	2.35405	28.302	3.58769	0.00605201
TCONS_00271547	SASH1	6:148558720-148873186	16.0977	1.36713	-3.55763	0.00335663
TCONS_00020947	DDAH1	1:85731456-86044046	1.76149	20.2906	3.52594	0.0372105
TCONS_00193475	TGOLN2	2:85545146-85555548	2.21071	24.8707	3.49186	0.0372105
TCONS_00088299	LGALS3	14:55590827-55612126	2.5356	28.131	3.47176	0.0359069
TCONS_00004639	MAST2	1:46252658-46501896	0.904814	9.65157	3.41507	0.0412828
TCONS_00073957	SLC38A1	12:46576838-46663800	7.93558	0.752308	-3.39894	0.0454058

Table of the 20 most differentially expressed novel transcripts, with log fold change (Log2_FC) between groups with Benjamini-Hochberg FDR corrected q-values. The list is sorted on Log2_FC.

Supplementary Table 6. A list of potentially significant GO (Biological process) terms according to mRNA sequencing results

GO ID	Term	Annotated	Significant	Expected	Rank in ClassicFisher	CI Fisher	ClassicKS	ElimKS
GO:0019083	Viral transcription	56	56	56	1	1	0.00088	0.00088
GO:0048596	Embryonic camera-type eye morphogenesis	12	12	12	2	1	0.00238	0.00238
GO:0045454	Cell redox homeostasis	15	15	15	3	1	0.00252	0.00252
GO:0009653	Anatomical structure morphogenesis	418	418	418	4	1	0.00166	0.00166
GO:0006413	Translational initiation	61	61	61	5	1	0.00311	0.00311
GO:0006809	Nitric oxide biosynthetic process	11	11	11	6	1	0.00317	0.00317
GO:0043624	Cellular protein complex disassembly	64	64	64	7	1	0.00336	0.00336
GO:0050792	Regulation of viral process	18	18	18	8	1	0.00406	0.00406
GO:0042558	Pteridine-comtaining compound metabolic process	3	3	3	9	1	0.00447	0.00447
GO:0051289	Protein homotetramerization	11	11	11	10	1	0.00469	0.00469
GO:0003333	Amino acid transmembrane transport	7	7	7	11	1	0.00496	0.00496
GO:0090257	Regulation of muscle system process	22	22	22	12	1	0.00525	0.00525
GO:0033157	Regulation of intracellular protein transport	41	41	41	13	1	0.00527	0.00527
GO:0007166	Cell surface receptor signaling pathway	472	472	472	14	1	0.00595	0.00595
GO:0051208	Sequestering of calcium ion	10	10	10	15	1	0.00598	0.00598
GO:0010557	Positive regulation of macromolecule biosynthetic process	254	254	254	16		0.00651	0.00651
GO:0002520	Immune system development	135	135	135	17	1	0.00695	0.00695
GO:0051173	Positive regulation of nitrogen compound metabolic process	253	253	253	18	1	0.00754	0.00754
GO:0071318	Cellular response to ATP	3	3	3	19	1	0.00762	0.00762
GO:0006414	Translational elongation	51	51	51	20	1	0.00851	0.00851

The top 20 significant GO terms for the genes found to be differentially expressed and their corresponding annotation for Biological process (BP).

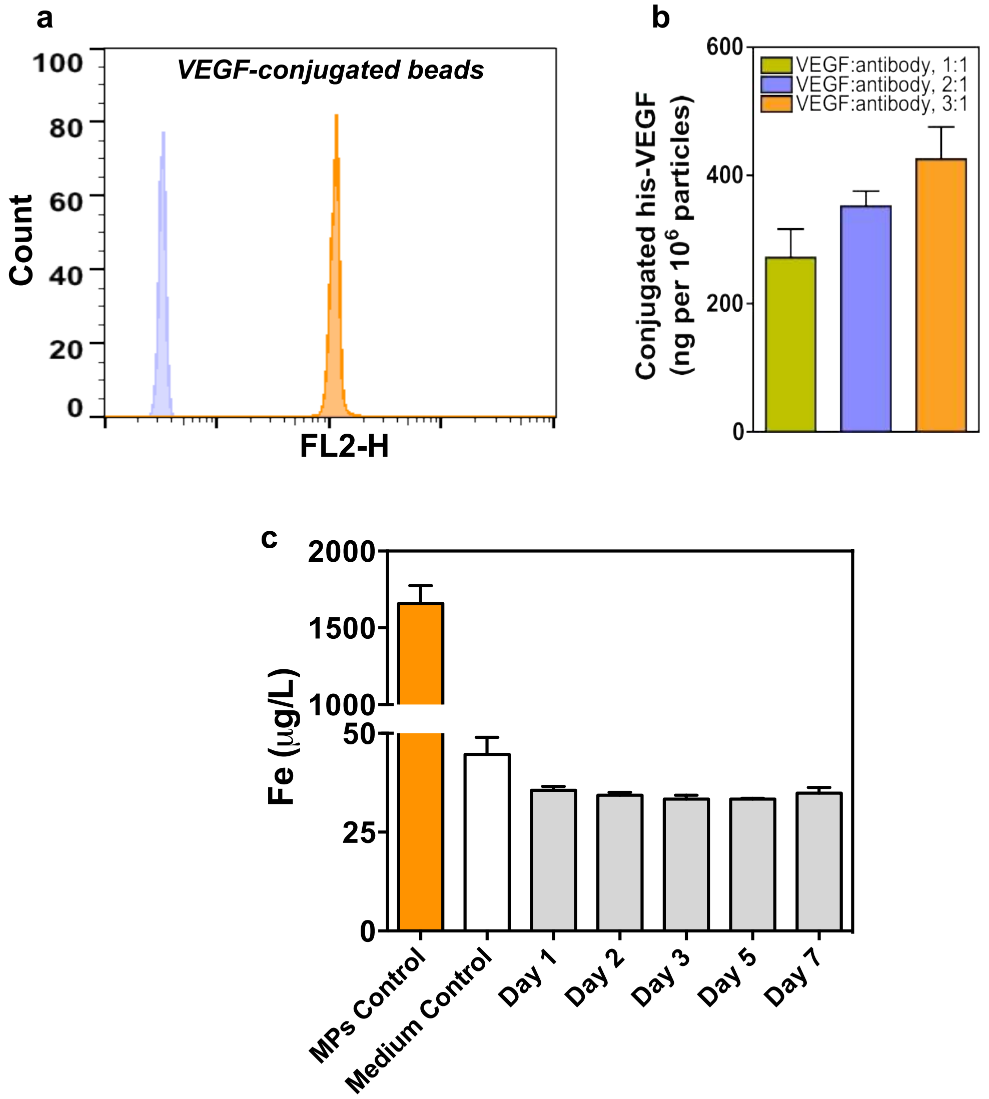

Supplementary Figure 1. Characterization of MPs. (a) Evaluation of VEGF conjugation onto MPs by flow cytometry. His-VEGF-conjugated MPs were stained with PE-conjugated anti-human VEGF antibody before flow cytometry characterization. Blue histogram indicates MPs without VEGF while orange histogram indicates VEGF-coated microparticles. (b) Amount of VEGF conjugated to microparticles: effect of the initial ratio of his-VEGF and anti-his-VEGF antibody. The immobilization of his-VEGF was quantified by ELISA measurements. Values are given as average ± SEM (n=4-8). (c) Release of iron from microparticles up to 7 days as determined by ICP-MS analyses. Microparticles (MPs Control) and EGM-2 (Medium Control) were used as controls. Values are given as average ± SEM (n=3).

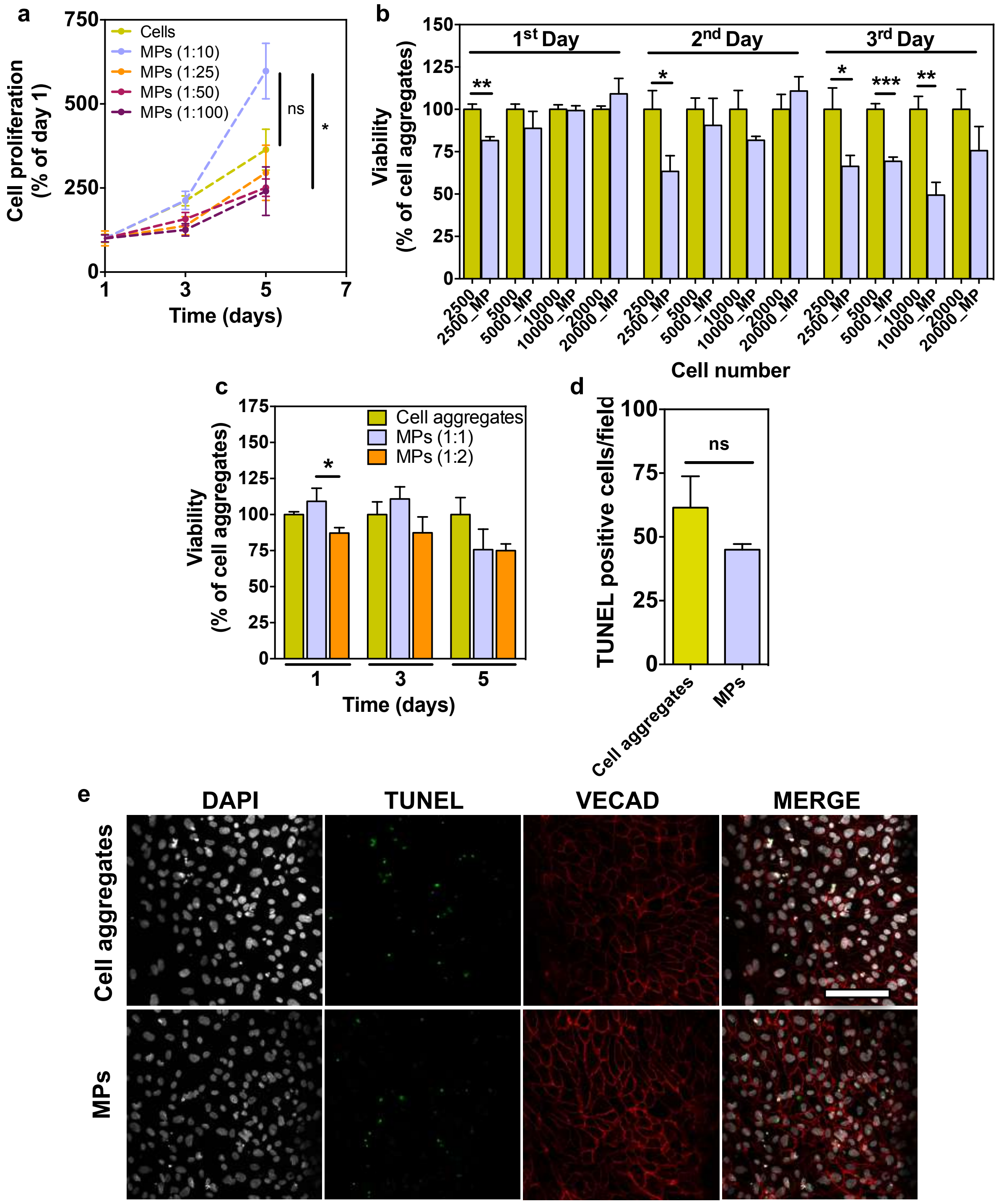

Supplementary Figure 2. MP cytotoxicity evaluation. (a) OEPCs cultured in monolayer were treated with different ratios of MPs (1:10, 1:25, 1:50, 1:100 cell:MP ratio) up to 5 days. Compared to cell aggregates, higher cell:MP ratios were used due to the fact that cell-bead interaction is lower in monolayer cultures. WST-1 was used to follow kinetics of cell proliferation. (b) Viability of OEPCs in cell aggregates having variable cell number. Different numbers of OEPCs (2,500-20,000 cells/aggregate) were seeded as hanging drops and the viability of the cells was evaluated up to 3 days. (c) Viability of OEPCs in cell aggregates having 1:1 and 1:2 cell:MP ratios. 20,000 cells were used per cell aggregate. (d) OEPC apoptosis as quantified by TUNEL assay. Cell aggregates (20,000 cells with a 1:1 cell:MP ratio) were cultured for 2 days before analyses. The number of TUNEL positive cells per field

was quantified. (e) Representative images of TUNEL staining. Scale bar corresponds to 50 µm. The values in (a-d) are given as average ± SEM for 4-6 different samples for each group. *$P \leq 0.05$ (*), $P \leq 0.01$ (**), $P \leq 0.001$ (***)*. In (a) and (c), one-way Anova followed by a Bonferroni post-test was used for statistical analyses. In (b) and (d), unpaired *t-test* was performed between cell aggregates and cell aggregates containing MPs.

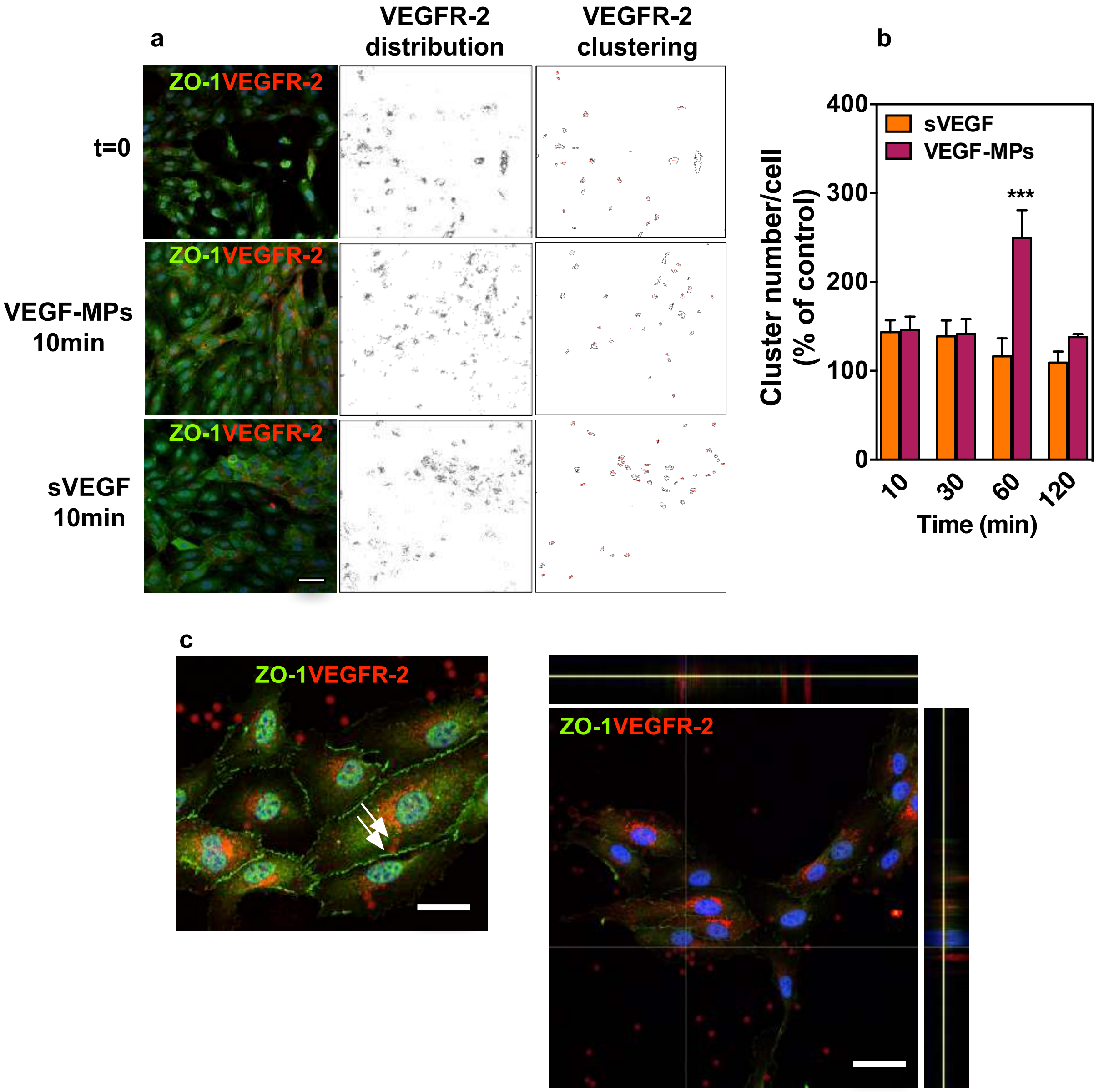

Supplementary Figure 3. VEGF-MPs internalization and VEGFR-2 clustering. OEPCs cultured in monolayer were treated with VEGF-MPs (1:2, cell:VEGF-MP ratio) or with same amount of sVEGF for different time points (i.e. 10, 30, 60, 120 min). VEGFR-2 clustering was quantified by confocal microscopy after cell fixation and immunostaining. (a) Representative confocal microscopy images of receptor clustering and analyses. Black and white images prepared by ImageJ were used for quantification of VEGFR-2 clustering. Time zero images were used to determine the threshold of particle size. Cell numbers in each image were calculated using DAPI stainings to normalize the results (cluster/cell). (b) Quantification of VEGFR-2 clustering. Values are average ± SEM. Images from 3 samples in each group were used in the analysis. Unpaired *t-test* was performed between sVEGF and VEGF-MPs groups for statistical analysis. $P \leq 0.001$ *(***)*. (c) Representative confocal microscopy images showing the internalization of VEGF-MPs after 2 h. OEPCs cultured in monolayer were treated with VEGF-MPs (1:2, cell:VEGF-MP ratio) for 2 h. VEGF-MPs internalization was quantified by confocal microscopy after cell fixation and immunostaining. Arrows indicate intracellular MPs. Scale bar corresponds to 20 μm.

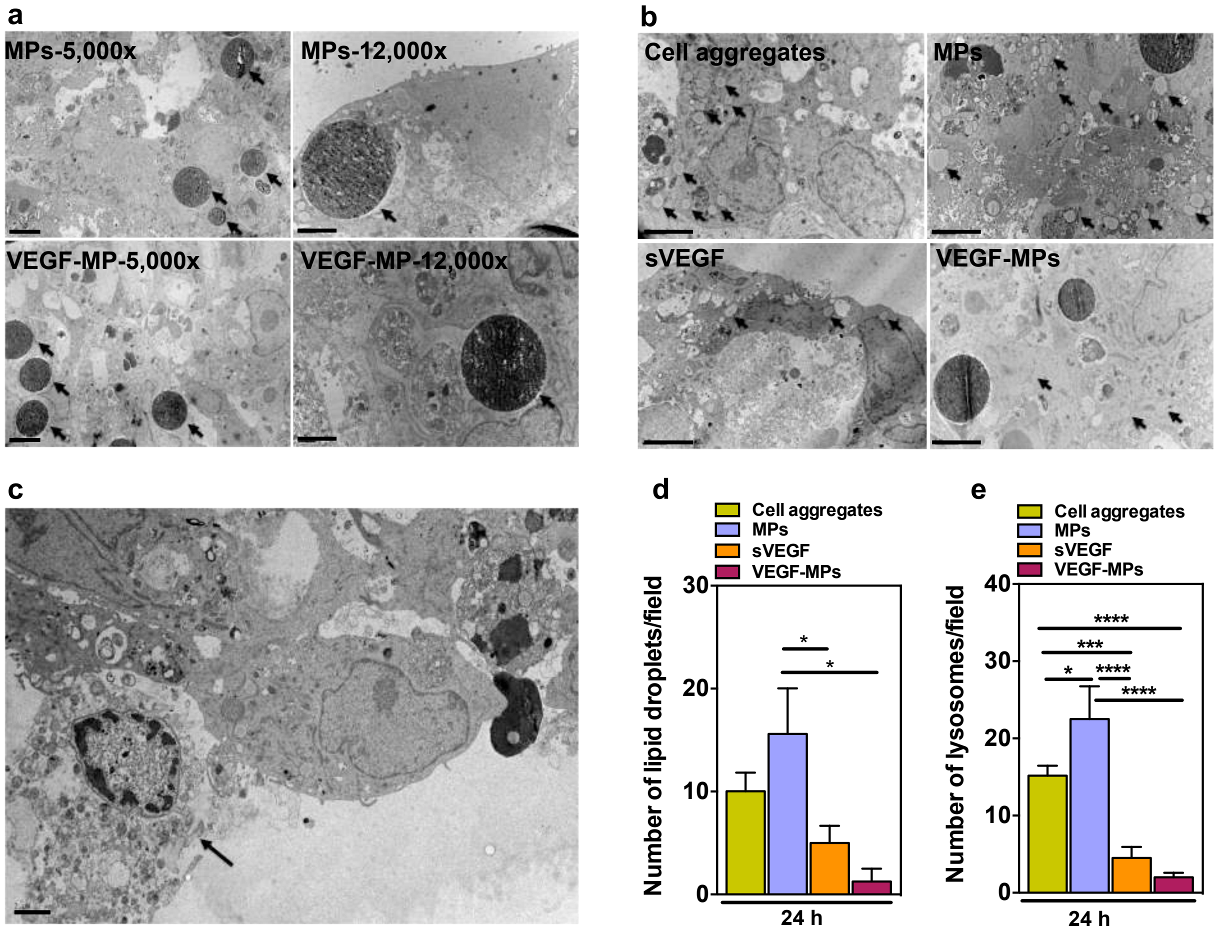

Supplementary Figure 4. Ultrastructural analysis of OEPC aggregates. (a) Transmission electron micrographs of OEPC aggregates containing blank MPs or VEGF-MPs showing the internalization of MPs by the cells after 24 h (Scale bars: 4 µm for 5,000× magnification; 2 µm for 12,000× magnification; arrows indicate MPs with the size of 4.5 µm). (b) The OEPC aggregates containing soluble (sVEGF) or immobilized VEGF (VEGF-MPs) show lower formation of cellular stress-related lipid droplets than control groups (cell aggregates) or blank MPs (Scale bars: 4 µm; magnification: 8,000×; time point: 24 h; arrows indicate lipid droplets). (c) Control group clearly shows necrotic cells in OEPC aggregates (Scale bar: 2 µm; magnification: 6,000×; time point: 24 h; arrow indicates necrotic cell in EC aggregate). (d-e) Quantification of lipid droplets (d) and lysosomes (e) in OEPC aggregates for different experimental conditions. The countings were performed using TEM photographs with 12,000× magnifications. Values are average ± SEM, n=3-8. For statistical analysis, one-way Anova followed by a Bonferroni post-test was used. $P \leq 0.05$ (*), $P \leq 0.01$ (**), $P \leq 0.001$ (***) and $P \leq 0.0001$ (****).

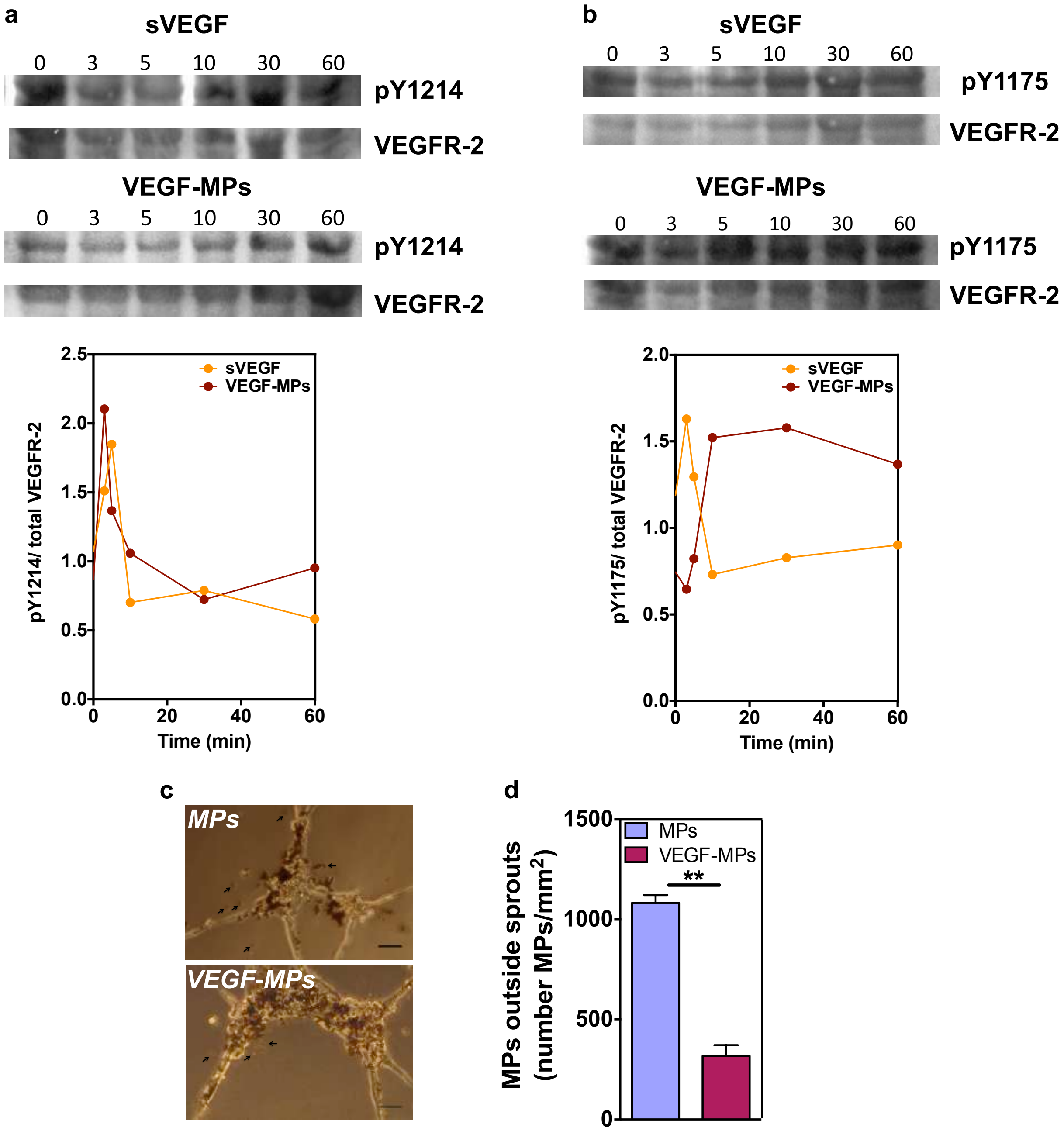

Supplementary Figure 5. Bioactivity of sVEGF and VEGF-MPs in OEPCs. (a-b) VEGFR-2 tyrosine phosphorylation induced by sVEGF or VEGF-MPs. OEPCs were aggregated up to 60 min and then lyzed. (c-d) VEGF-MPs and MPs interaction with OEPCs when seeded on Matrigel. (c) VEGF-MPs strongly interact with OEPC cord-like structures on Matrigel while blank MPs tend to aggregate and not to interact with cells. Arrows show the MPs that are not interacting with cells. Bar corresponds to 50 µm. (d) Quantification of non-interacting MPs. Values are average ± SEM, n=2 images/group were used for the calculations. The statistical analysis was performed using unpaired *t-test*. *P ≤ 0.01 (**)*.

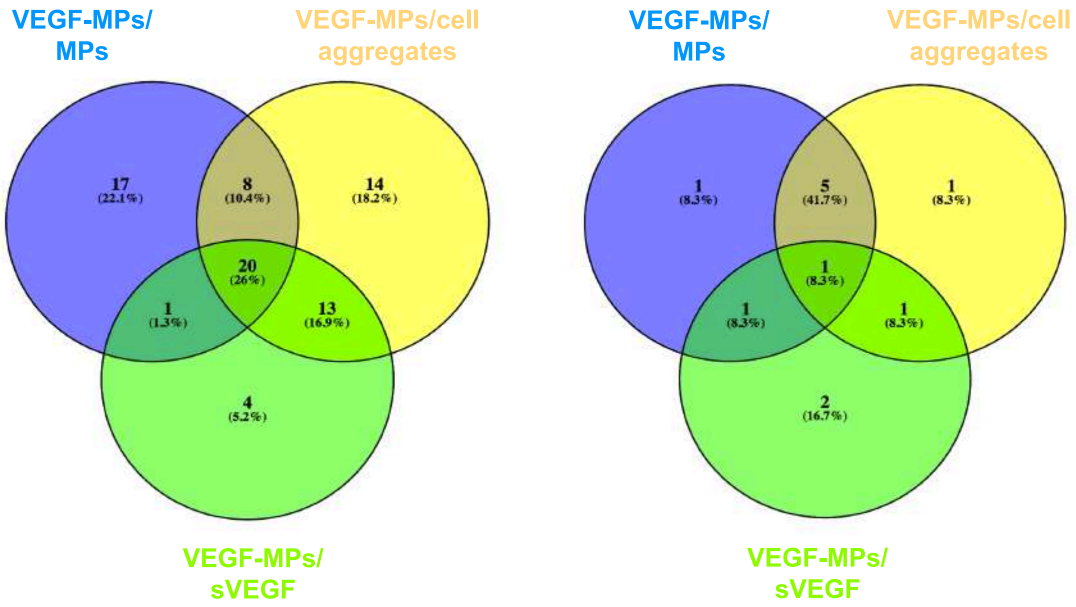

Common downregulated miRNAs: hsa-miR-217 hsa-miR-216a, hsa-miR-503, hsa-miR-31, hsa-miR-100, hsa-miR-24, hsa-miR-23a, hsa-miR-27a, hsa-miR-30a, hsa-miR-151-5p, hsa-miR-29c, hsa-miR-20a, hsa-miR-17, hsa-miR-106b, hsa-let-7f, hsa-miR-21, hsa-miR-126, hsa-miR-374a, hsa-let-7g, hsa-miR-126*

Common upregulated miRNAs: hsa-miR-576-5p

Supplementary Figure 6. miRNA microarray analysis. The common miRNAs downregulated or upregulated in VEGF-MP group compared with cell aggregates, MPs and sVEGF experimental groups were determined using VENNY software and presented in the figure.

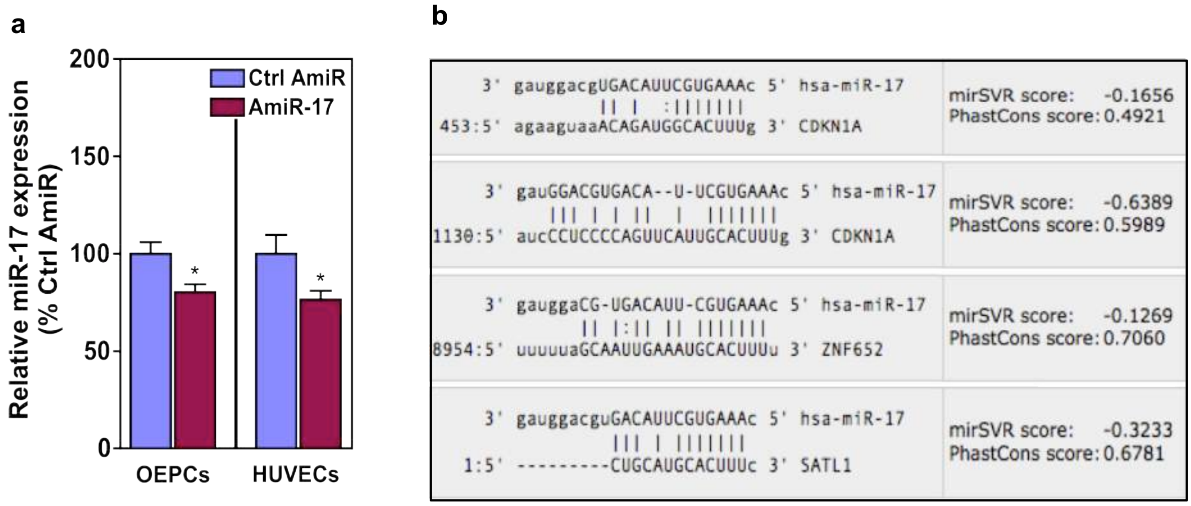

c

Gene ID	Gene	Locus	Control AntagomiR FPKM	AntagomiR-17 FPKM	Log2 FC	q value
TCONS_00052415	EIF4G2	11:10804859-10830657	6.11352E-97	88.4983	326.083	0.0494118
XLOC_036358	GRM7	3:6532165-7783215	0.124649	3.28693	4.7208	0.000675141
XLOC_024345	ZNF652	17:47366567-47447360	2.74285	14.3267	2.38496	0.000675141
XLOC_061942	SATL1	X:84343441-84364054	1.01125	5.03149	2.31484	0.0314161
XLOC_045782	CDKN1A	6:36555310-36932613	106.49	124.417	0.224474	0.633159

Supplementary Figure 7. Evaluation of *miR-17* downregulation and *miR-17* gene targets. (a) Confirmation of *miR-17* downregulation in OEPCs and HUVECs treated with *antagomiR-17* for 48 h. Results are average ± SEM, n=4. The statistical analysis was performed using unpaired *t-test*. *P ≤ 0.05 (*)*. The results were normalized to the control antagomiR condition for each cell type. (b) Interaction of *miR-17* with its gene targets determined by sequencing data in our system (Info taken from the database: www.microrna.org [1]). (c) Top transcripts upregulated after the transfection with *antagomiR-17*, which are the targets of *miR-17*, according to the mRNA sequencing data (n=3).

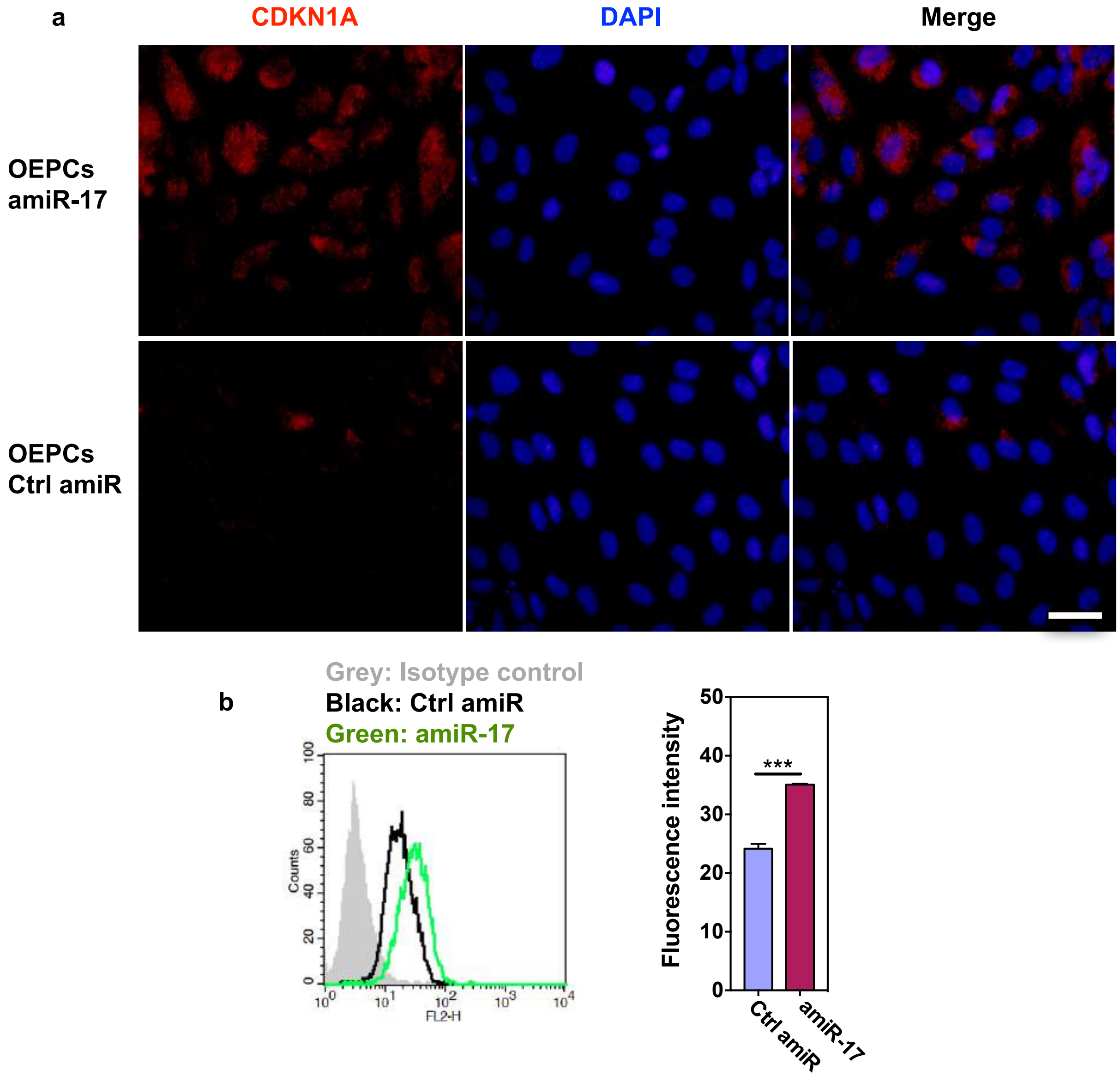

Supplementary Figure 8. CDKN1A expression in OEPCs after transfection with *amiR-17*. OEPCs were transfected with 50 nM *amiR-17* or 50 nM control amiR for 48 h. After additional incubation in hypoxia (0.1% O_2) for 24 h, they were stained with mouse anti-human CDKN1A antibody. (a) Fluorescence microscopy results. CDKN1A increased at protein level for OEPCs transfected with *amiR-17*. Scale bar corresponds to 20 µm. (b) Flow cytometry results. Results are average ± SEM, n=3. For statistical analysis, unpaired *t-test* was performed. $P \leq 0.001$ *(***)*.

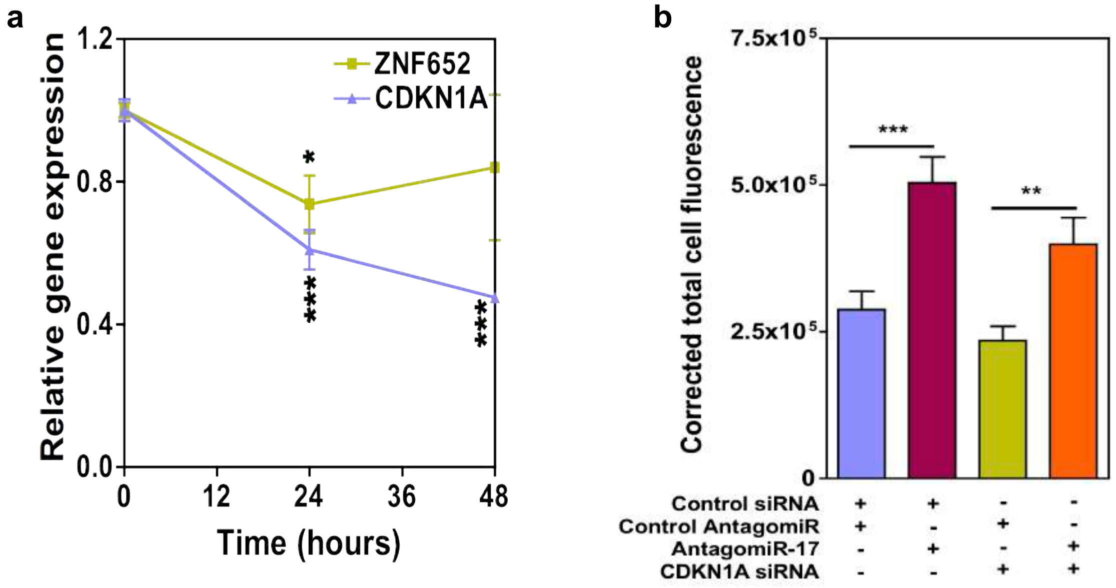

Supplementary Figure 9. Silencing of *miR-17* targets. (a) qRT-PCR results confirm the downregulation of gene targets (i.e. *ZNF652* and *CDKN1A*) after the treatment with siRNAs against human *ZNF652* and *CDKN1A*. (b) Quantification of CDKN1A silencing at protein level (results obtained by image analyses of confocal microscopy images). Corrected total cell fluorescence was calculated according to the equation: Integrated density − (Area of selected cell × Mean fluorescence of background readings). Results were given as average ± SEM. n=4 in (a) and n=3 in (b). Unpaired *t-test* was used for statistical analysis. *$P \leq 0.05$ (*), $P \leq 0.01$ (**), $P \leq 0.001$ (***).*

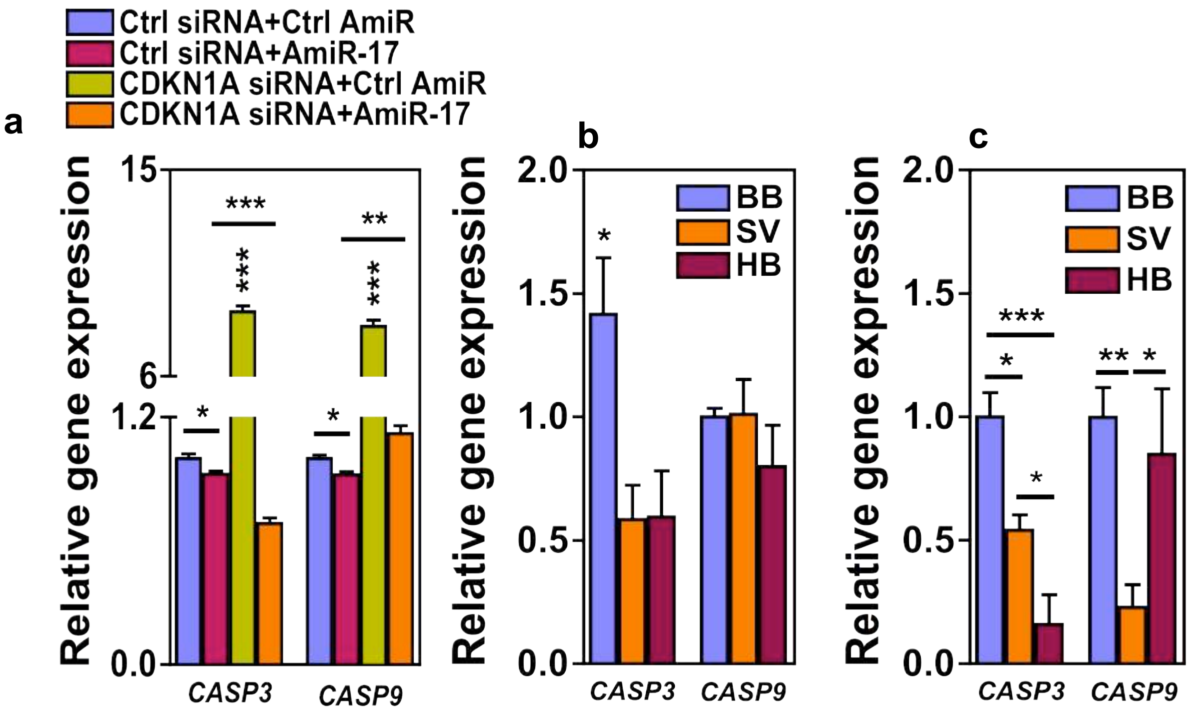

Supplementary Figure 10. Expression of *CASP3* and *CASP9* transcripts in OEPCs. (a) Silencing of gene target *CDKN1A* by siRNA for 48 h showed an increase in the expression of *CASP3* around 10 times and *CASP9* around 8 times. (b-c) Conjugated VEGF decreases the expression of *CASP3* and *CASP9* both *in vitro* (b) and *in vivo* (c) (subcutaneous injections, after 24 h). U6 was used to normalize the results. Values are given as average ± SEM, n=4. For statistical analysis, one-way Anova followed by a Bonferroni post-test was used. $P \leq 0.05$ (*), $P \leq 0.01$ (**) and $P \leq 0.001$ (***). The asterisks on CDKN1A siRNA+Ctrl AmiR group (***) in (a) indicate that in this group, the increase in *CASP3* and *CASP9* is statistically significant ($P \leq 0.001$) compared with all other groups in the graph.

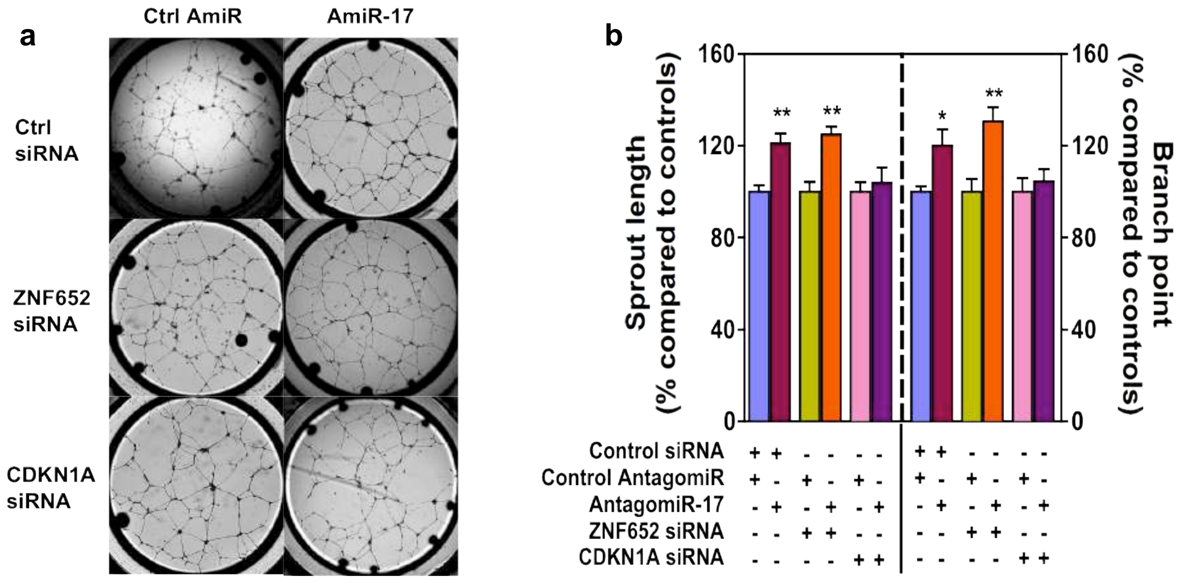

Supplementary Figure 11. Silencing of *miR-17* target *CDKN1A* reduces sprouting of OEPCs after treatment with *amiR-17*. OEPCs were double-transfected with 40 nM siRNAs (i.e. control siRNA, *ZNF652* siRNA or *CDKN1A* siRNA) and 50 nM antagomiRs (control amiR or *amiR-17*) for 48 h and incubated under hypoxia conditions (0.1% O_2) in starvation medium (EGM-2 without VEGF and with 1% FBS). (a) Representative light microscopy images of all experimental groups. (b) Effect of silencing *ZNF652 or CDKN1A* in the angiogenic effect of *amiR-17*. *ZNF652* siRNA-treatment did not impair angiogenesis potential of *amiR-17*, while *CDKN1A* siRNA-treatment decreased significantly the effect of *amiR-17*. The values are normalized to their control groups and given as average ± SEM (n=5). Unpaired *t-test* was used for statistical analysis. *$P \leq 0.05$ (*), $P \leq 0.01$ (**)*. The sprout photos were taken using IN Cell Analyzer from GE Healthcare with 2× objective and analyzed in ImageJ.

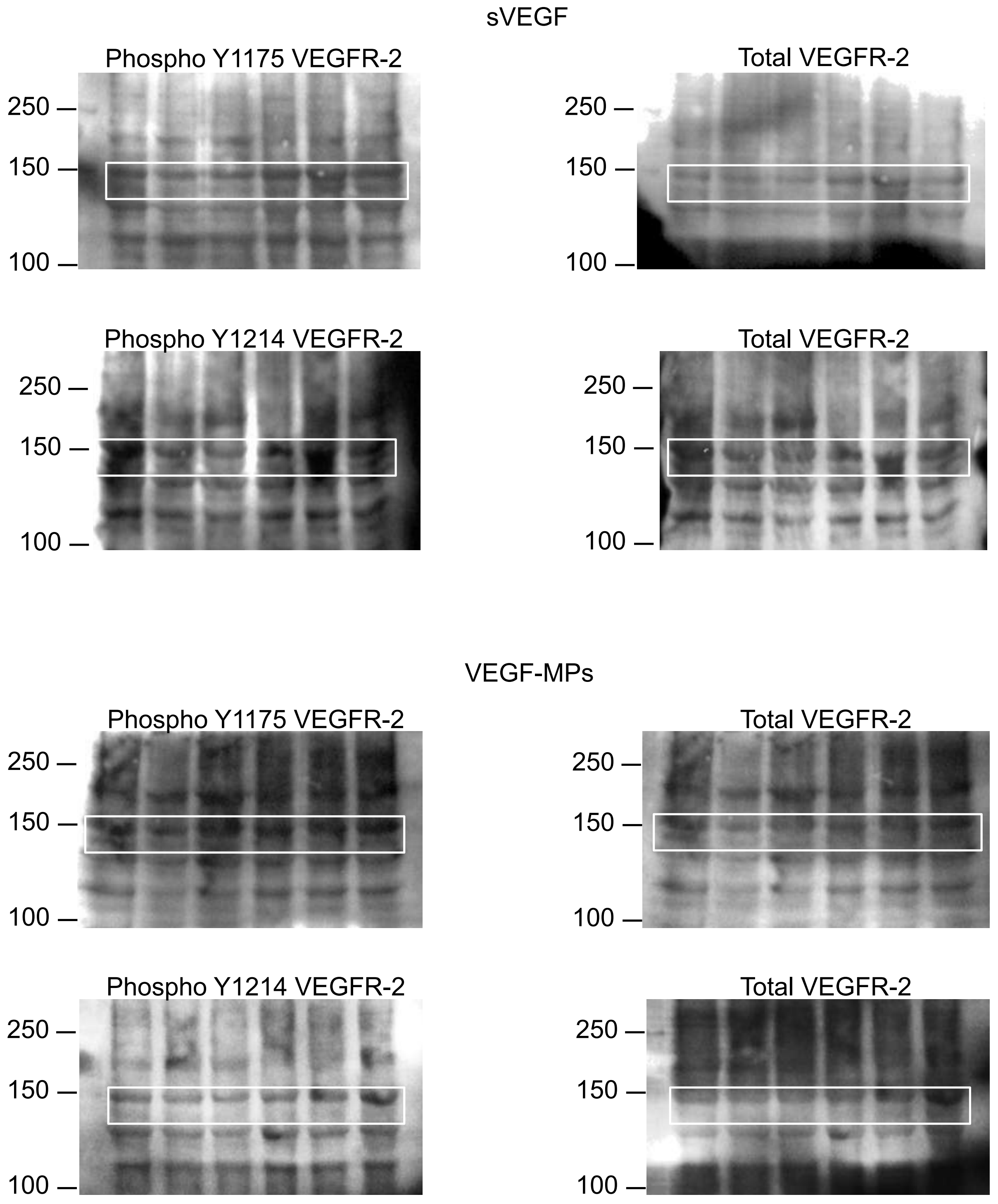

Supplementary Figure 12. Full gel images for WB. Uncropped images of the gels for Supplementary Fig. 5a-b. White rectangles indicate the bands used for the calculations.

Supplementary Reference

1. Betel D, Wilson M, Gabow A, Marks DS, Sander C. The microRNA.org resource: targets and expression. *Nucleic Acids Res* **36**, D149-153 (2008).
